# Developing a Neural–Kalman Filtering Approach for Estimating Traffic Stream Density Using Probe Vehicle Data

**DOI:** 10.3390/s19194325

**Published:** 2019-10-07

**Authors:** Mohammad A. Aljamal, Hossam M. Abdelghaffar, Hesham A. Rakha

**Affiliations:** 1 Charles E. Via, Jr. Department of Civil and Environmental Engineering, Center for Sustainable Mobility, Virginia Tech Transportation Institute, Virginia Tech, Blacksburg, VA 24061, USA; 2 Department of Computers Engineering and Systems, Engineering Faculty, Mansoura University, Mansoura, Dakahlia 35516, Egypt or; 3 Center for Sustainable Mobility, Virginia Tech Transportation Institute, Virginia Tech, Blacksburg, VA 24061, USA

**Keywords:** real-time estimation, probe vehicle, traffic density, neural network, level of market penetration rate

## Abstract

This paper presents a novel model for estimating the number of vehicles along signalized approaches. The proposed estimation algorithm utilizes the adaptive Kalman filter (AKF) to produce reliable traffic vehicle count estimates, considering real-time estimates of the system noise characteristics. The AKF utilizes only real-time probe vehicle data. The AKF is demonstrated to outperform the traditional Kalman filter, reducing the prediction error by up to 29%. In addition, the paper introduces a novel approach that combines the AKF with a neural network (AKFNN) to enhance the vehicle count estimates, where the neural network is employed to estimate the probe vehicles’ market penetration rate. Results indicate that the accuracy of vehicle count estimates is significantly improved using the AKFNN approach (by up to 26%) over the AKF. Moreover, the paper investigates the sensitivity of the proposed AKF model to the initial conditions, such as the initial estimate of vehicle counts, initial mean estimate of the state system, and the initial covariance of the state estimate. The results demonstrate that the AKF is sensitive to the initial conditions. More accurate estimates could be achieved if the initial conditions are appropriately selected. In conclusion, the proposed AKF is more accurate than the traditional Kalman filter. Finally, the AKFNN approach is more accurate than the AKF and the traditional Kalman filter since the AKFNN uses more accurate values of the probe vehicle market penetration rate.

## 1. Introduction

Real-time traffic state estimates have been increasingly recognized following the introduction of recent advanced technologies such as connected vehicle (CV) technologies. CVs aim to improve road safety by potentially reducing human errors, mitigating traffic congestion levels by offering alternative routes, and reducing on-road emissions and fuel consumption [1]. Nowadays, conducting research with limited probe vehicle data (e.g., CVs) is a challenge, especially when no additional data sources are provided. Hence, past research has utilized probe data in conjunction with existing detection systems to enhance proposed traffic models, despite the limitation that fixed detection techniques (e.g., loop detectors) always have some noise in their data [2,3,4].

A probe vehicle is defined as a vehicle that provides real-time information, such as its instantaneous position and speed. Several benefits of using probe vehicle data have been recognized; for example, the high quality of data compared with existing data sources (e.g., cameras and loop detectors), and data can be collected at any location inside the network, thus offering a clear picture about traffic behavior at any time. Therefore, transportation agencies are putting effort into facilitating the use of probe vehicle data.

Limited studies have used only information from probe vehicle data (e.g., Global Positioning Systems [GPSs]) to estimate the state of on-road traditional vehicles [5], such as traffic travel time, traffic density, traffic speed, and traffic volume. The real-time estimation of traffic density is important to achieving better traffic operations management in urban areas. This paper aims to estimate the total number of vehicles on signalized link approaches using only probe vehicle data. The estimate outcomes can be provided to traffic signal controllers to optimally determine the allocation of green time for each traffic signal phase [6,7], leading to better intersection performance measures such as intersection delays and vehicle crashes [8,9]. One concern with using probe vehicles is measuring their level of market penetration (LMP). The LMP is defined as the ratio of the total number of probe vehicles to the total number of vehicles. Providing accurate LMP estimates improves the estimation accuracy of the vehicle counts [5]. Therefore, in this paper, a machine-learning technique is developed to provide reliable LMP estimates.

## 2. Related Work

Different statistical tools have been used to estimate the total number of vehicles on arterial roads and freeways, such as the Kalman filter (KF) [10], Bayesian statistics [11], and Particle filter [12] approaches. The literature shows the benefits of using the KF technique in addressing different aspects of the traffic estimation problem. The KF has been used to estimate the traffic travel time [13,14], traffic speed [15,16], and traffic density [5,17]. Different detection techniques have been employed to estimate the number of vehicles, such as loop detectors, camera systems, and probe data. Two loop detectors, one at the entrance and the other at the exit of the link, are utilized to measure the total number of arrivals and departures, then the number of vehicles are simply obtained by applying the flow continuity equation [18]. A robust KF model with at least three loop detectors on the tested link was employed to estimate the number of vehicles on the link in [17]. The study derived the KF state equation from the flow continuity equation, while the measurement equation was derived from the relationship of the detector time occupancy and space occupancy; however, the cost of implementing such an algorithm in the field is high given the number of sensors needed. Another study employed the KF to estimate the number of vehicles on multi-section freeways. The state equation was derived from the flow continuity equation, while the measurement equation was derived from the hydrodynamic relationship between traffic speed and density [19]. Loop detectors were used in addition to speed sensors in the middle of the tested section. However, the proposed algorithm is hard to employ in the field due to the high cost of implementation. A video record, another detection technique, was used to estimate the traffic density for signalized links [20]. In that study, the authors used the space-mean speed rather than the traffic flow in the state equation due to high errors accompanied with sensor failures. Their argument takes into account that the space-mean speed is taken as an average quantity while the traffic flow is a cumulative quantity. They also demonstrated the importance of having knowledge about the system noise characteristics to improve the performance of the KF model. Consequently, the authors of this paper applied an adaptive Kalman filter (AKF) to enable real-time estimates of statistical parameters of the system noise rather than using predefined values for the entire simulation (as assumed in the traditional KF model).

As illustrated in the literature, stationary sensors, such as loop detectors and camera systems, suffer from poor detection accuracy and have high installation and maintenance costs. Advanced detection techniques such as GPS data have proven to be more accurate without the need to install additional hardware. Consequently, recent studies have developed several traffic estimation models using fusion data (combination of two different data sources) to estimate the number of vehicles with the aim of achieving better accuracy than using only one source of data. In many of the works using fusion data, the KF technique was employed for estimating traffic density. One study achieved accurate estimated traffic density results using the traffic flow values measured from a video detection system and the travel time obtained from vehicles equipped with GPS devices [2]. The proposed estimation approach in this study differs in two significant ways from the proposed AKF model, namely only probe vehicle data are used with a variable time interval rather than a fixed value (the updating time interval was 1 min in [2]), and the proposed estimation approach uses the AKF to allow for real-time estimates of statistical parameters of the state and measurement noise.

Reviewing the literature, the KF model has proven its ability to address estimation research problems for different traffic applications. However, it is hard to implement in real-world applications due to hard estimates of statistical characteristics of the system noise (mean and variance). Consequently, researchers have developed the AKF to solve this issue and make field implementation possible. Chu et al. proposed an AKF model to estimate freeway travel time using both loop detectors and probe data [21]. They presented the estimation method for noise statistic parameters that was proposed in [22]. This estimation method of statistical parameters is known for its simplicity in handling errors and its fast processing time. Hence, in this study, the estimation of the statistical parameters uses the same estimation procedure as in Chu et al.’s study. It should be noted that the main difference between the proposed estimation approach and Chu et al.’s approach is that our model uses only probe vehicle data.

In a recent study, the KF model was proposed to estimate the number of vehicles on signalized link approaches using only probe vehicle data [5]. The KF state equation was based on the traffic flow continuity equation and thus one value of probe vehicle LMP (ρ), for the entire link, is used to scale up the probe measurements to reflect the total flow in the second term of the flow continuity equation as presented in Equation (1). It was found that using two LMP values (at the entrance and the exit of the link) produce more accurate vehicle count estimates, especially when dealing with low LMPs, as described later in Section 4.3. In Equation (1), N(t) is the number of vehicles traversing the link at time (*t*), Δt is the variable duration of the updating time interval, N(t−Δt) is the number of vehicles traversing the link in the previous interval, $q^{in}$ and $q^{out}$ are the probe flows entering and exiting the link between (t−Δt) and (*t*), respectively, and ρ is the LMP of probe vehicles.

(1) $$N\left(t\right)=N\left(t-\Delta t\right)+\frac{\Delta t}{\rho }\left[q^{in}\left(t\right)-q^{out}\left(t\right)\right]$$

Machine learning has proven its ability to provide accurate estimates for different traffic characteristics [23,24,25,26,27,28]. Traffic speed and density have been estimated using an artificial neural network (ANN) model [23]. Video and Bluetooth data were used to build the ANN model. The traffic flow data were manually extracted from the video records, while the speed data were constructed from the collected Bluetooth travel time data. The neural network model (NN) is able to address the research problem if a good quantity of training data is accessible. Another study conducted several machine learning techniques such as k-means clustering, k-nearest neighbor classification, and locally weighted regression to estimate traffic speed [24] using archived data of speeds, counts, and densities. They found that machine learning models can improve the accuracy of speed estimation. Khan et al. [25] used artificial intelligence to classify the level of service in a freeway segment based on traffic density values. They used loop detectors and CV data to develop support vector machine and k-nearest neighbor classification. Results indicated higher accuracy from the support vector machine algorithm than the k-nearest neighbor classification algorithm. Estimating hourly traffic volumes between sensors was addressed using an NN model in the Maryland highway network [27], deploying both probe vehicles and automatic traffic recording station data to construct the NN model. A comparison was also made between linear regression, k-nearest neighbor, support vector machine with linear kernel, random forest, and NN models, concluding that the NN model performed the best. The proposed approach produced 24% more accurate estimates than current volume profiles.

In this research study, an AKF technique was applied to estimate real-time vehicle counts along signalized link approaches using only probe vehicle data. The study then considers the recommendation of Aljamal et al’s study [5] by using two LMP values at the entrance and the exit of the tested link. To achieve this task, an NN model was developed to provide real-time estimates of the LMP values to improve the accuracy of the proposed AKF model. After that, the paper develops the new AKFNN approach after combining the AKF with the developed NN models. The proposed study extends the state-of-the-art in vehicle count estimates by making four major contributions:

- The study tests the proposed AKF model using only probe vehicle data. The approach was evaluated considering different probe vehicle LMPs ranging from 10% to 90% at increments of 10%.
- The study develops an NN model to estimate the LMP of probe vehicles at the exit of the link to reflect the total vehicle departures.
- The study tests the developed AKFNN approach by using a fusion of probe and single-loop detector data. A comparison between the traditional KF, AKF, and AKFNN models is presented.
- The study examines the impact of the initial conditions on the AKF estimation model. Three initial condition parameters are tested: the initial vehicle count estimate, the initial mean estimate of the state noise errors, and the a priori initial covariance of the state system.

This paper is organized as follows. The first section describes the development of the simulation data. The second section describes the estimation models and the problem formulation for the KF, AKF, and AKFNN models. The third section discusses the results of the new proposed models. The fourth section provides the conclusions of the study and recommended future work.

## 3. Development of Simulation Data

This paper relies on the INTEGRATION traffic simulation model [29] to validate and test the accuracy of the proposed models. The INTEGRATION software has been extensively validated and demonstrated to replicate empirical observations [30,31,32,33,34,35]. Specifically, INTEGRATION was used to create synthetic data for conditions not observed in the field to quantify the sensitivity of the proposed method to the link length and traffic demand level. The selected tested link is located in downtown Blacksburg, Virginia, with an approximate length of 102 m based on ArcGis software, and connects two signalized intersections. The link characteristics were calibrated to local conditions using typical values, which included a free-flow speed of 40 (km/h), a speed-at-capacity of 32 (km/h), a jam density of 160 (veh/km/lane), and a base saturation flow rate of 2100 (veh/h/lane), which resulted in a roadway capacity of 700 (veh/h) given the cycle length and green times of the traffic signal. The traffic signal cycle length is 75 s and it has four phases with the following displayed green times: 5, 25, 5, and 28 s. The tested link here is assigned with a displayed green time of 25 s. These values were consistent with what was coded in the field.

The INTEGRATION simulation model was used to ease the generation of probe vehicle data as real probe data are not easy to access. For each LMP, a total of 50 scenarios were generated with different random seeds as conducted in [25]. Forty-nine scenarios were used to train and validate the proposed NN model, and scenario number 50 was considered the testing data set. The INTEGRATION model generates a “time-space” file which provides some information about the probe vehicles during their trips for every second. The time-space file records the instantaneous position, speed, and spacing for each probe vehicle. In addition to that, a loop detector is installed at the entrance of the tested link to create a detector output file which provides some data about the simulation behavior such as speed, traffic volume, and occupancy at the detection location.

## 4. Estimation Models

This section first summarizes some crucial points regarding estimating the vehicle count as discussed in the authors’ last research study [5]. In addition, this section describes the proposed AKF estimation model for estimating the vehicle count along signalized link approaches, and demonstrates the difference of the state-of-the-art KF model in [5] and the new proposed AKF model. Finally, an NN model is developed to provide estimates of the probe vehicle LMPs to be used in the proposed AKF model equations to attain higher accuracy. Two vehicle count estimation models are described in this section: (1) the AKF model, which uses only probe vehicle data; and (2) the AKFNN model, which fuses probe and single-loop detector data. The single-loop detector data were mainly used to develop the NN model.

### 4.1. Summary of the Developed KF Model

In a previous study [5], the authors developed a KF model to produce reliable vehicle count estimates using only probe vehicle data. In that study, the authors introduced a novel variable estimation time interval as opposed to the traditional fixed time interval. The estimation time interval was defined as the time when exactly *n* probe vehicles traversed the tested link. It was proven that the variable time interval, compared to a fixed time interval (e.g., 20 s), led to improved estimation accuracy. An illustrative example to show the benefits of using the variable time interval. if the approach’s LMP is 10%, the number of probe vehicles will obviously be low. If we treat the problem using a fixed estimation interval, then the probability of observing zero probe vehicles within an interval will be high for short estimation time intervals, making the estimation inefficient and inaccurate. Accordingly, low LMPs require long intervals (e.g., 300 s) to ensure that at least one probe vehicle is on the approach. In contrast, approaches with high LMPs can use short estimation intervals (e.g., 20 s). Consequently, treating the estimation time interval as a variable produces an efficient and convenient way of determining the duration of the estimation period. For more details, readers may refer to [5].

One concern about the KF model is the use of predefined fixed values of the statistical parameters, mean and variance, of the KF state and measurement errors. Applying the KF model in real-world problems is limited since the statistical parameters are assumed to be known [21]. The mean and variance entities are known as variable rather than fixed values. To produce a flexible model, this study employs the AKF model to provide real-time estimates of the statistical parameters of the KF state and measurement errors as described in the following section.

### 4.2. Adaptive Kalman Filter (AKF)

The traditional KF technique is utilized with predefined error values of the state and measurement noise; these error values remain constant for the entire simulation. However, these values are hard to obtain in the field and they are always changing with time. Hence, an AKF is developed to overcome this issue and to dynamically estimate the error values in the state and measurement estimates. The AKF is comprised of two equations: (a) state equation and (b) measurement equation. The state equation is derived from the traffic flow continuity equation as defined in Equation (2). The state equation computes the number of vehicles by continuously adding the difference in the number of vehicles entering and exiting the section to the previously computed cumulative number of vehicles traveling along the section. This integral results in an accumulation error which requires fixing, and thus the measurement equation is needed. In Equation (2), the ρ value can be observed from historical data.

(2) $$N\left(t\right)=N\left(t-\Delta t\right)+\frac{\Delta t}{\rho }\left[q^{in}\left(t\right)-q^{out}\left(t\right)\right]$$

The state equation produces accurate results if the scaled traffic flows $(q^{in}/\rho_{in}$ and $q^{out}$/$\rho_{out})$ are accurate [5], as shown in Section 4.3. The total counts can be extracted from traditional loop detectors or video detection systems. We should note here that the ρ value in Equation (2) plays a major role in delivering accurate outcomes. ρ is defined as the ratio of the number of probe vehicles $\left(N_{probe}\right)$ to the total number of vehicles $\left(N_{total}\right)$, as shown in Equation (3). For instance, if ρ is equal 0.1, and the number of probe vehicles is 5, then the expected total number of vehicles is 50.

(3) $$\rho =N_{probe}/N_{total}$$

Equation (4) describes the hydrodynamic relationship between the macroscopic traffic stream parameters (flow, density, and space-mean speed),

(4) $$q=ku_{s}$$

where *q* is the traffic flow (vehicles per unit time), *k* is the traffic stream density (vehicles per unit distance), and $u_{s}$ is the space-mean speed (distance per unit time). The $u_{s}$ can be represented as shown in Equation (5),

(5) $$u_{s}=D/TT$$

where *D* is the link length and TT is the average vehicle travel time. Since probe vehicles can share their instantaneous locations every Δ*t*, the travel time of each probe vehicle can be computed for any road section. Thus, the probe vehicle travel time is used in the measurement equation, using Equations (4) and (5). The measurement equation can be written as shown in Equation (8):

(6) $$TT\left(t\right)=D\;\times \;\frac{k(t)}{\bar{q}\left(t\right)}$$

(7) $$TT\left(t\right)=\frac{1}{\bar{q}}\left[k\left(t\right)\times D\right]=\frac{1}{\bar{q}\left(t\right)}N\left(t\right)$$

(8) $$TT\left(t\right)=\;H\;\left(t\right)\times N\left(t\right)$$

where $\bar{q}$ is the average traffic flow entering and exiting the link, and H(t) is a transition vector that converts the vehicle counts to travel times, and is the inverse of the average flow (i.e., the first term of Equation (7)), as shown in Equation (9).

(9) $$H\left(t\right)=\frac{1}{\bar{q}\left(t\right)}=\frac{2\times \rho }{q^{in}\left(t\right)+q^{out}\left(t\right)}$$

The system state and measurement equations can be written as in Equations (10) and (11), considering the errors (noise). The term u(t) is the given inputs for the system. The vector H(t) is used to convert the vehicle counts to travel times. The vector w(t−Δt) is the state noise and is assumed to be Gaussian noise with the mean of m(t) and variance of M(t). The measurement noise v(t) is assumed to be Gaussian noise with the mean of r(t) and variance of R(t).

(10) State Equation:     N(t)=N(t−Δt)+u(t)+w(t−Δt)

$$u\left(t\right)=\frac{\Delta t}{\rho }\left[q^{in}\left(t\right)-q^{out}\left(t\right)\right]$$

(11) $$\mathrm{Measurement}\;\mathrm{Equation}:\;\mathrm{TT}\left(t\right)=\;H\;\left(t\right)\times N\left(t\right)+v\left(t\right)$$

$$H\left(t\right)=\frac{1}{\bar{q}\left(t\right)}=\frac{2\times \rho }{q^{in}\left(t\right)+q^{out}\left(t\right)}$$

The proposed AKF estimation model can be solved using the following equations:

(12) $${\hat{N}}^{-}\left(t\right)={\hat{N}}^{+}\left(t-\Delta t\right)+u\left(t\right)+m\left(t-\Delta t\right)$$

(13) $${\hat{P}}^{-}\left(t\right)=\;{\hat{P}}^{+}\left(t-\Delta t\right)+M\left(t-\Delta t\right)$$

(14) $$G\left(t\right)=\;{\hat{P}}^{-}\left(t\right)H{\left(t\right)}^{T}\;{\left[H\left(t\right){\hat{P}}^{-}\left(t\right)\;H{\left(t\right)}^{T}+R\left(t\right)\right]}^{-1}$$

(15) $${\hat{N}}^{+}\left(t\right)={\hat{N}}^{-}\left(t\right)+G\left(t\right)\;\left[TT\left(t\right)-H\left(t\right){\hat{N}}^{-}\left(t\right)-r\left(t\right)\right]$$

(16) $${\hat{P}}^{+}\left(t\right)={\hat{P}}^{-}\left(t\right)\times \left[1-H\;\left(t\right)\;G\;\left(t\right)\right]$$

where ${\hat{N}}^{-}$ is the a priori estimate of the vehicle counts calculated using the measurement prior to instant *t*, and ${\hat{P}}^{-}$ is the a priori estimate of the covariance error at instant *t*. The Kalman gain (*G*) is demonstrated in Equation (14). The posterior state estimate (${\hat{N}}^{+}$) and the posterior error covariance estimate (${\hat{P}}^{+}$) are updated as shown in Equations (15) and (16), considering the probe vehicle travel time measurements. In the next section, the estimation steps of the noise statistical parameters (m,M,r,R) are described.

#### 4.2.1. Online Estimation of Noise Statistics

An online estimate is conducted to optimally find the errors in the state and the measurement variables, to make the KF more efficient and applicable in real-world applications. As pointed out in the literature, the traditional KF assumes predefined errors in the system, which is not the case in real applications. A set of unknown noise statistical parameters, (m,M,r,R), needs to be estimated at every estimation step. The online estimate procedure follows the same procedure presented in [21].

The mean (*m*) and variance (*M*) of the state noise are shown in Equations (17) and (18), respectively.

(17) $$m=\frac{1}{n}\sum_{t=1}^{n}\;m(t),\;\mathrm{where}\;m\left(t\right)={\hat{N}}^{+}\left(t\right)-{\hat{N}}^{+}\left(t-\Delta t\right)-u(t)$$

(18) $$M=\frac{1}{n-1}\sum_{t=1}^{n}\;\left[\left(m(t)-m\right).{\left(m(t)-m\right)}^{T}-\left(\frac{n-1}{n}\right){\hat{P}}^{+}\left(t-\Delta t\right)-{\hat{P}}^{+}\left(t\right)\right]$$

where m(t) is the state noise at time *t*, the first term of Equation (18) is the covariance of w at time *t*, n is the number of state noise samples.

The mean (*r*) and variance (*R*) of the measurement noise are shown in Equations (19) and (20), respectively.

(19) $$r=\frac{1}{n}\sum_{t=1}^{n}r(t),\;\mathrm{where}\;r\left(t\right)=TT\left(t\right)-H\left(t\right)\;{\hat{N}}^{-}\left(t\right)$$

(20) $$R=\frac{1}{n-1}\sum_{t=1}^{n}\;[\left(r(t)-r\right).{\left(r(t)-r\right)}^{T}-\left(\frac{n-1}{n}\right)H\left(t\right){\hat{P}}^{-}\left(t\right)H^{T}\left(t\right)]$$

where R(t) is the observation noise at time *t*. The first term of Equation (20) is the covariance of v at time *t*, and n is the number of measurement noise samples. As a summary, the KF and AKF models use the same equations except for the fact that the AKF model estimates the statistical parameters of the noise for every estimation step using Equations (17) to (20).

As found in our previous study [5], providing the system equations real-time estimates of $\rho_{in}$ and $\rho_{out}$ should improve the estimation accuracy. In this study, a single-loop detector was installed at the entrance of the tested link to produce real-time estimates of $\rho_{in}$. In contrast, in the next section, an NN model is developed to obtain real-time estimates for the $\rho_{out}$ values.

### 4.3. Neural Network

NN is a machine learning technique that aims to recognize relationships between vast amounts of data by employing a certain number of neurons in every single hidden layer to achieve better accuracy [36]. The network consists of three main layers: the input layer, the hidden layer, and the output layer. This section takes into account the recommendation of using two market penetration rates (at the entrance and exit of the link) rather than one market penetration rate along the tested link in the KF equations [5]. Accordingly, the state equation and the H vector in the measurement equation are revised as presented in Equations (21) and (22). $\rho_{in}$ and $\rho_{out}$ are the probe LMP at the entrance and the exit of the link, respectively.

(21) $$N\left(t\right)=N\left(t-\Delta t\right)+\Delta t\left[\frac{q^{in}\left(t\right)}{\rho_{in}\left(t\right)}-\frac{q^{out}\left(t\right)}{\rho_{out}\left(t\right)}\right]$$

(22) $$H\left(t\right)=\frac{1}{\bar{q}\left(t\right)}=\frac{2}{\frac{q^{in}\left(t\right)}{\rho_{in}\left(t\right)}+\frac{q^{out}\left(t\right)}{\rho_{out}\left(t\right)}}$$

**Figure 1:**
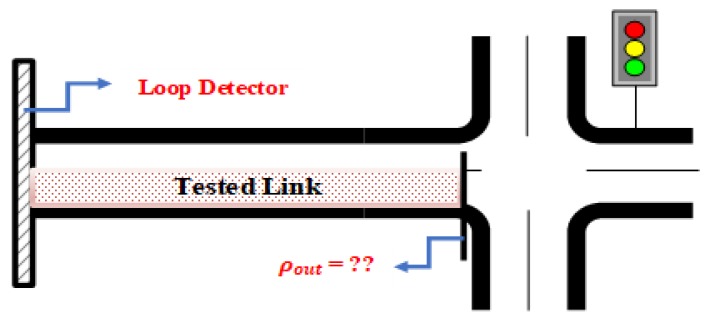
Tested link.

A single-loop detector was installed at the entrance of the link to measure $\rho_{in}$ and also to use as an input to the NN model. Accordingly, this study develops an NN model to estimate $\rho_{out}$. The tested link is shown in Figure 1. The next section describes the selected inputs (features) and the output variables of the NN model.

#### Characteristics of the NN: Input and Output Variables

Previous research has used different features to build machine learning models [23,24,25,26]. Fusing video and Bluetooth data was used to estimate traffic density and speed. The traffic flow was manually extracted from the video records, while the speed data were constructed from the collected Bluetooth travel time data [23]. Another study relied on archived data of traffic speeds, counts, and density to estimate traffic speed [24]. Distance headway, number of stops, and speed data were identified as useful features to achieve accurate density estimates [25]. They employed loop detectors and CV data. In a recent study, Sekula et al. used probe and automatic traffic recording station data to extract the features of the NN model [27]. The selected features were the (1) speed of probe vehicles, (2) weather data such as temperature, visibility, precipitation, and weather status, (3) infrastructure data (speed limits, number of lanes, class of the road, and type of the road), (4) temporal data such as the day of the week, and (5) volume profiles based on historical data. The literature showed that the traffic speed is always used as a model feature, especially when probe vehicle data are used. In contrast, the traffic flow is always used when stationary sensors (e.g., loop detector) are used.

In this paper, a fusion of probe and single-loop detector data is utilized to produce the model features. The single-loop detector was installed at the entrance of the link and thus $\rho_{in}$ can be computed directly using Equation (3). The $\rho_{out}$ variable is calculated from the NN (the NN output). Seven possible inputs (features) were considered in the NN model, as defined in Table 1. Conducting a feature selection technique to validate the importance of each feature for the NN model, the number of the model features was dropped to five features. It should be noted that the selected model inputs can be easily extracted when probe vehicles are on the link. $\rho_{out}$ can be expressed as a function of the selected inputs, as presented in Equation (23).

(23) $$\rho_{out}=f\left(A_{t},A_{p},u_{s},S_{1},S_{2}\right)$$

The $\rho_{out}$ values vary between 0 and 1, the 0 value means that no probe vehicles were observed at the exit of the link, while the value of 1 means that the $D_{p}$ value is the same as the $D_{t}$. The selected inputs must be relevant to the model output $\rho_{out}$ to allow the NN model to build a strong relationship between the model inputs and outputs, and therefore produce high estimation accuracy. For instance, in our case, the $\rho_{out}$ value decreases as $A_{t}$ and $A_{p}$ increase. For instance, a high value of $A_{t}$ means that the link is more congested and thus the number of departures ($D_{t}$) is expected to be high. The $\rho_{out}$ value also decreases with increasing speed ($S_{1}$, $S_{2}$, and $u_{s}$). The speed is an indicator of the congestion level of the link; for instance, if the speed is low, then more vehicles are expected to be on the link, leading to higher values of $D_{t}$.

**Figure 2:**
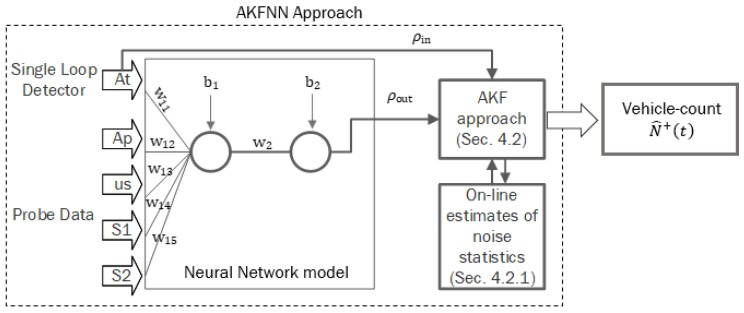
Flowchart for adaptive Kalman filter with a neural network (AKFNN) approach.

A single hidden layer with one neuron, with a transfer function of hyperbolic tansgent sigmoid, was used to build the NN model as shown in Figure 2. The Levenberg–Marquardt (LM) optimization has been proven in the literature to outperform the gradient decent and conjugate gradient methods for medium-sized problems [37]. Furthermore, the LM is considered the fastest back-propagation algorithm and thus was implemented in the proposed approach. The weights and biases of the developed NN model are described below. $w_{1}$ depicts the weights between the input layer and the hidden layer, while $w_{2}$ represents the weight between the hidden layer and the output layer. b1 and b2 represent the biases at the hidden and output layers, respectively. Figure 2 describes the proposed AKFNN approach, combining the AKF model with the NN model.

$$w_{1}=\left[0.43\;0.19\;-47.28\;0.36\;-0.43\right],\;w_{2}=\left[1.70\right],\;b1=\left[-46.62\right],\;b2=\left[0.95\right]$$

## 5. Results

This section evaluates the performance of the proposed models. The first subsection evaluates the performance of the AKF model and then compares the AKF with the KF model (Section 5.1). The second subsection presents the performance of the NN model used for estimating the LMP of probe vehicles at the exit of the link ($\rho_{out}$) (Section 5.2). The third subsection compares the performance of AKF with the AKFNN approach (Section 5.3). The fourth subsection investigates the sensitivity of the AKF estimation model to the initial conditions (Section 5.4). The accuracy of the proposed models was evaluated based on the root mean square error (RMSE) as shown in Equation (24). The RMSE has been frequently used in the literature to measure the difference between the model estimates and the actual values.

(24) $$\mathrm{RMSE}\;(\mathrm{veh})\;=\;\sqrt{\sum_{t=1}^{n}[{\hat{N}}^{+}\left(t\right)-N\left(t\right){]}^{2}/n}$$

where ${\hat{N}}^{+}\left(t\right)$ represents the estimated vehicle count values, N(t) represents the actual vehicle count values, and *n* is the total number of estimations. All simulation scenarios start with the following initial conditions: an initial vehicle count estimate of zero (${\hat{N}}^{+}\left(0\right)=0\;\mathrm{veh}$), which is the same value of the actual vehicle count, and initial mean and the prior covariance estimates of the state system (m(0) = 2 veh and ${\hat{P}}^{-}\left(0\right)$ = 75 veh^2^) if the LMP scenario is less than or equal 60%, and (m(0) = 9 veh ${\hat{P}}^{-}\left(0\right)$ = 120 veh^2^) if the LMP scenario is greater than 60%. The proposed models were evaluated using different probe vehicle LMPs, including 10%, 20%, 30%, 40%, 50%, 60%, 70%, 80%, and 90%. For each scenario, a Monte Carlo simulation was conducted to create 300 random samples of probe vehicles from the full data set.

### 5.1. Comparison of the KF and the AKF Models

This section evaluates the proposed AKF model with real-time estimates of the error statistical parameters for the state and the measurement. This section also compares the proposed AKF model with the developed KF model in [5], as shown in Table 2. Results show that the AKF outperforms the KF model in most scenarios except for the scenarios with high LMPs (i.e., LMP of 80% and 90%). Results demonstrate the need to provide real-time estimates for the mean and variance error values in the state and measurement when dealing with low/medium LMPs. This happened due to high error in the fixed ρ value that was used, which then produced high error in the vehicle count estimate. The AKF improved the traditional KF vehicle-count estimation accuracy by up to 29%. In contrast, for high LMPs, the user may proceed with predefined statistical values for the state and measurement (mean and variance error values), due to low errors in the vehicle count estimates (low error in the ρ value). In conclusion, a simple KF can be used with high LMPs without the need to change statistical noise parameters at every estimation step.

### 5.2. Developed NN Model

**Figure 3:**
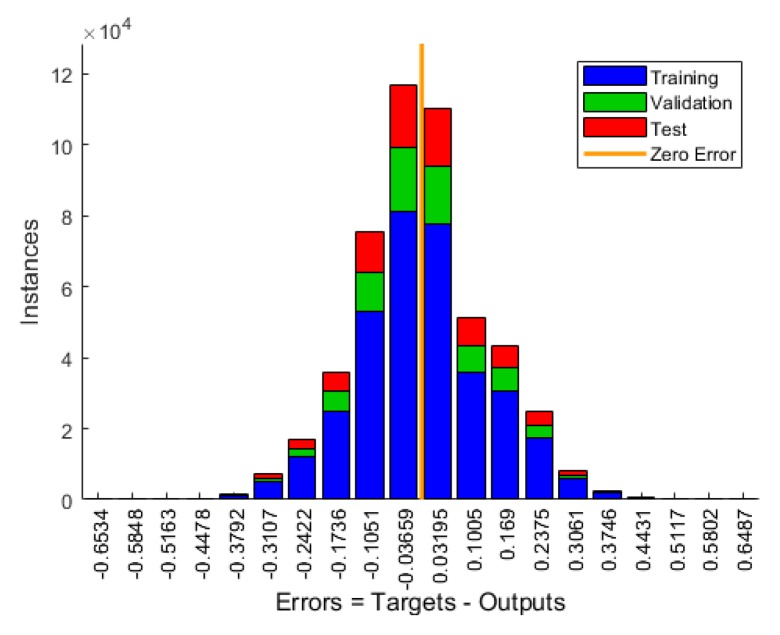
Error histogram for the training, validation, and testing data set.

**Figure 4:**
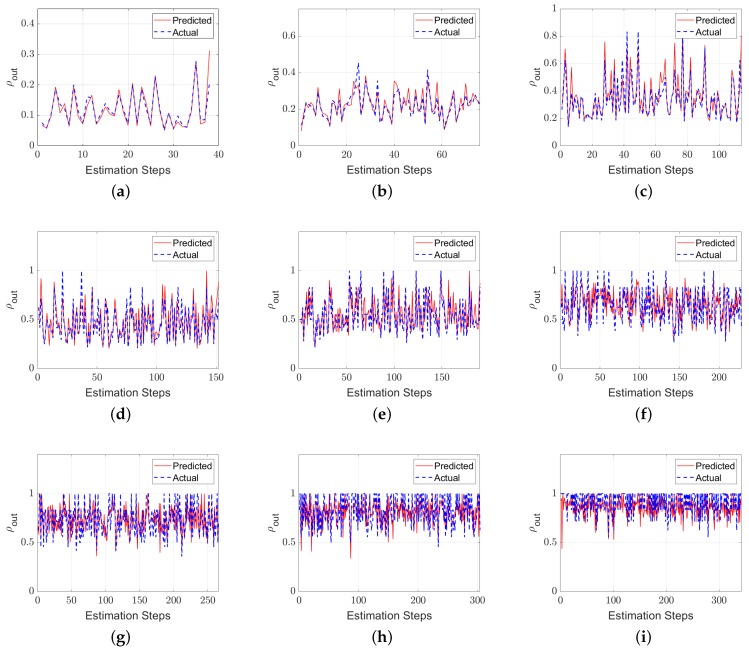
Actual and estimated values of $\rho_{out}$ for different level of market penetration (LMP) scenarios: (**a**) 10%, (**b**) 20%, (**c**) 30%, (**d**) 40%, (**e**) 50%, (**f**) 60%, (**g**) 70%, (**h**) 80%, and (**i**) 90% LMP.

The NN model was employed to predict the ($\rho_{out}$) value, which is used to reflect the total number of vehicle departures from the given number of probe vehicle departures. The data set was divided into 70% for training, 15% for validation, and 15% for testing. The validation data set is used to measure network generalization and to avoid any over fitting problems [38]. The developed NN performance is shown in Table 3. The mean square error (MSE) is 0.01 and the R value is close to 1.0. The R value measures the correlation between model outputs and desired outputs. A value close to 1.0 means that the model outputs are very close to desired outputs. Figure 3 shows the error histogram for the training, validation, and testing data and their deviations from the zero error bar. Most of the errors lie around the zero error bar, which means that the developed NN model appropriately addressed the research goal (i.e., estimating $\rho_{out}$). Figure 4 presents the predicted and actual values for the $\rho_{out}$ at different LMPs.

### 5.3. Comparison of the AKF and the AKFNN Models

This section demonstrates the impact of using two ρ values rather than using one predefined ρ value. The average predefined ρ value is defined as the value for the entire tested link. The average ρ value remains constant for the entire simulation for each LMP scenario. For instance, if the scenario of 10% LMP is tested, the ρ value in both the state and measurement is treated as a value of 0.1. In this study, the authors proposed the use of two ρ values; one at the entrance and one at the exit of the link to reflect the total number of arrivals and departures from the given total number of probe arrivals and departures, respectively.

$\rho_{in}$ is measured directly using the installed loop detector at the entrance of the link. The developed NN model is used to predict the $\rho_{out}$ values (Section 5.2). Then, the $\rho_{in}$ and $\rho_{out}$ values are utilized in the AKF equations. Recall that the AKF model relies only on probe vehicle data, while the AKFNN model uses a fusion of probe vehicle and single-loop detector data.

**Figure 5:**
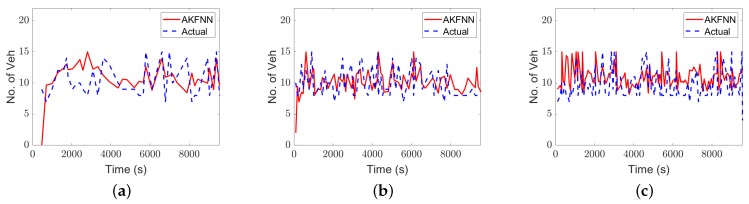

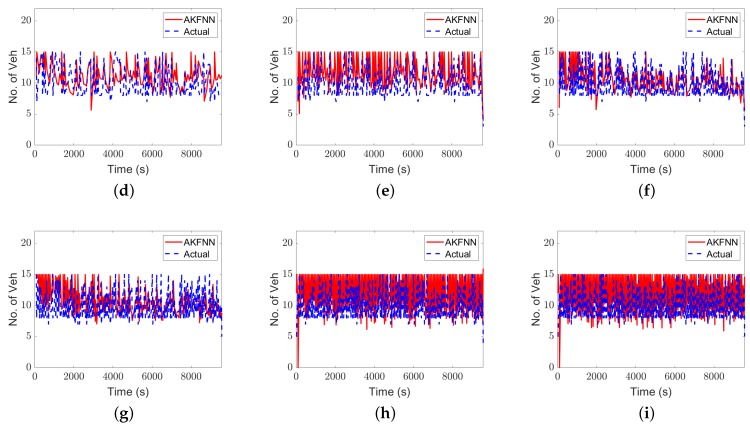
Actual and estimated vehicle counts over estimation intervals for different LMP scenarios: (**a**) 10%, (**b**) 20%, (**c**) 30%, (**d**) 40%, (**e**) 50%, (**f**) 60%, (**g**) 70%, (**h**) 80%, and (**i**) 90% LMP.

In Table 4, the RMSE values using the AKF and the AKFNN models are presented. The results demonstrate the benefits of using the AKFNN approach rather than the AKF approach, where the estimation accuracy is improved by up to 26%. This finding proves what was recommended by Aljamal et al.’s previous study [5] to consider two ρ values rather than one value. As a result, the proposed AKFNN approach is robust and produces reasonable errors even with low LMPs. For instance, the estimated vehicle count values are off by 3.7 veh when the LMP is equal to 10%. Figure 5 presents the vehicle count estimation for different LMPs using the proposed AKFNN Approach.

### 5.4. Impact of the Initial Conditions on the AKF Model

The KF model, traditional and adaptive, is sensitive to the initial condition parameters, such as the posterior state estimate ($N_{i}$ = ${\hat{N}}^{+}\left(0\right)$), the mean of state noise ($m_{i}$ = m(0)), and the prior error covariance estimate ($P_{i}$ = ${\hat{P}}^{-}\left(0\right)$). These parameters are tuned by a trial-and-error technique to find the best initial condition values for seeking better KF estimation outcomes. However, in real applications, trial-and-error is not realistic and not easy to achieve. Hence, this section investigates the impact of initial conditions on the accuracy of the vehicle count estimation.

#### 5.4.1. Impact of Initial Estimate of the Vehicle Count ($N_{i}$)

**Figure 6:**
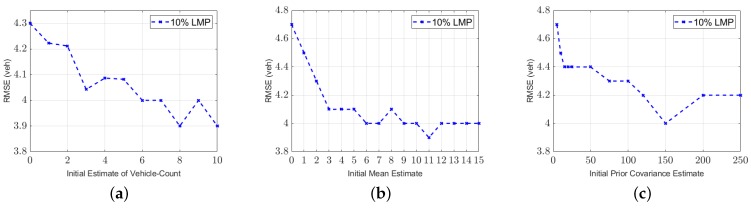
Impact of the initial conditions on the AKF model: (**a**) Initial estimate values $N_{i}$, (**b**) Initial mean estimate values $m_{i}$, and (**c**) Initial covariance estimate values $P_{i}$.

For the initial estimate value of the vehicle count ($N_{i}$), different values were evaluated (ranges from 0 to 10 at increments of 1). In this study, remember that all simulation scenarios start with an initial estimate of zero ($N_{i}=0\;\mathrm{veh}$), which is the same value as the actual vehicle count. Figure 6a presents the RMSE values for different $N_{i}$ values for the scenario of 10% LMP. As shown in the figure, the values of 8 and 10 produce the lowest RMSE. The RMSE value is equal to 4.3 veh when $N_{i}$ is equal to 0. In contrast, theRMSE value is equal to 3.9 veh when $N_{i}$ is equal to 8. As a result, starting the AKF model with the best initial estimate (e.g., $N_{i}$ = 8 veh) would reduce the errors and therefore improve the estimation accuracy.

#### 5.4.2. Impact of Initial Mean Estimate of the State System ($m_{i}$)

Another critical initial parameter in the AKF model is $m_{i}$. This parameter represents the mean value of the noise in the state equation. This paper tests 16 different $m_{i}$ values (i.e., 0, 1, 2, 3, 4, 5, 6, 7, 8, 9, 10, 11, 12, 13, 14, and 15). Figure 6b presents the vehicle count estimation RMSE values for different $m_{i}$ values. The RMSE value is equal to 4.7 veh when the simulation starts with a 0 value of $m_{i}$. In contrast, the RMSE value is 3.9 veh when the value of $m_{i}$ is equal to 11.

#### 5.4.3. Impact of Initial Prior Covariance Estimate of the State System ($P_{i}$)

The last parameter tested in this study is the initial prior estimate of error covariance $P_{i}$. The error covariance parameter describes the accuracy of the state system. For instance, if the covariance value is low, then the state outcome is accurate and close to the actual value. As stated in the literature, the initial parameters should always be tuned to achieve accurate estimation accuracy. Thirteen different $P_{i}$ values were tested (i.e., 5, 10, 15, 20, 25, 50, 75, 100, 120, 150, 200, and 250). Figure 6c presents the RMSE values using different $P_{i}$ values. The $P_{i}$ value of 150 veh^2^ produces the lowest RMSE values.

The research presented in this study evaluates the proposed approaches as they should be in real-world applications. Therefore, the trial-and-error technique was avoided since it is not a valid solution in the field. However, it was noticed that previous research always tunes the initial parameters to determine the best initial conditions when testing their estimation approaches [2,3,17]. If that is the case, let us assume that the proposed AKFNN approach always starts with the best initial value of $P_{i}$, which would produce less errors. Table 5 presents the RMSE when considering the trial-and-error technique (Tuned AKFNN). The AKFNN and the Tuned AKFNN approaches used the same values of $N_{i}$ and $m_{i}$, but they used different $P_{i}$ values. $N_{i}$ is assumed to be zero, while $m_{i}$ has two values based on the tested scenario: a value of 2 veh when low LMP scenarios are tested (LMP <= 60%), and a value of 9 veh with high LMP scenarios (LMP > 60%). From the table, tuning the $P_{i}$ value significantly improves the estimation accuracy for all scenarios (by up to 27%). For instance, at 10% LMP, the estimation error dropped from 3.7 to 3.3 vehicles. On the other hand, the estimated vehicle count values are off by 2.8 vehicles instead of 3.6 vehicles for the scenario of 20% LMP.

In conclusion, the AKF model was proven to be very sensitive to the initial conditions ($N_{i},m_{i},P_{i}$). Hence, starting the simulation with good assumptions of the initial conditions can significantly improve the estimation accuracy, as shown in Table 5. Finally, Table 6 presents the performance of the models discussed in the paper.

## 6. Conclusions

The research proposed a novel AKF model for estimating the number of vehicles on signalized approaches using only probe vehicle data. An AKF model was developed to provide real-time estimates of the statistical properties (mean and variance) for the state and measurement errors. The state equation is derived from the traffic flow continuity equation, while the measurement equation is constructed using the traffic hydrodynamic equation. Results show that the proposed AKF model outperforms the traditional KF model (improves the estimation accuracy by up to 29%), demonstrating the need to use real-time values of the statistical noise parameters in the KF model.

Two estimation models were presented, namely (a) the AKF and (b) the AKFNN. The AKF model uses only probe vehicle data assuming a fixed LMP value that is obtained from historical data, while the AKFNN uses a fusion of probe and single-loop detector data with real-time estimates of the LMP values ($\rho_{in}$ and $\rho_{out}$). In this paper, a robust NN model was developed to provide accurate real-time estimates of the $\rho_{out}$ values. The selected features of the NN model are $A_{t}$ (observed from the single-loop detector), $A_{p}$, $u_{s}$, $S_{1}$, and $S_{2}$ (observed from probe vehicles).

The AKF and the NN models were combined to develop the novel AKFNN approach. Results demonstrate that the AKFNN approach significantly improves the vehicle count estimation accuracy since the $\rho_{in}$ and $\rho_{out}$ values are estimated better. Subsequently, the paper compared the AKF with the AKFNN models, showing that the AKFNN model outperforms the AKF model, enhancing the estimation accuracy by up to 26%.

Finally, the study investigated the impact of the initial conditions ($N_{i}$, $m_{i}$, and $P_{i}$) on the AKF performance. Results show that the AKF model is very sensitive to the initial conditions. For instance, starting the simulation with an $N_{i}$ value of 8 instead of 0 improves the estimation accuracy by 10%. In addition, starting the simulation with an $m_{i}$ value of 11 instead of 2 enhances the estimation accuracy by up to 10%. For the $P_{i}$ parameter, an improvement of 7% could occur if the simulation starts with an initial value of 150 instead of 75 veh^2^. The study also tested the accuracy of the AKFNN estimation by allowing the $P_{i}$ parameter to be tuned (Tuned AKFNN approach), showing that more improvement could be achieved. Specifically, the Tuned AKFNN improves the accuracy by up to 27%.

In conclusion, both models (AKF and AKFNN) produce high estimation accuracy when compared with the state-of-the-art KF model. Proposed future work entails testing traffic signal performance using the estimates of the total number of vehicles as inputs to the traffic signal controller.

## Figures and Tables

**Table 1:** Definition of the NN model inputs.

Input Symbol	Definition	Unit
$A_{t}$	Total number of arrivals obtained from the single-loop detector	veh
$A_{p}$	Total number of probe arrivals	veh
$D_{p}$	Total number of probe departures	veh
$S_{1}$	Average speed for probe vehicles at link entrance	km/h
$S_{2}$	Average speed for probe vehicles at link exit	km/h
$u_{s}$	Space-mean speed for probe vehicles	km/h
$u_{t}$	Time-mean speed for probe vehicles	km/h

**Table 2:** Root mean square error (RMSE) values using the Kalman filter (KF) and the adaptive Kalman filter (AKF) models.

LMP (%)	RMSE (veh)		
	KF	AKF	Improvement (%)
10	6.0	4.3	29
20	5.6	4.0	28
30	5.0	3.8	23
40	4.6	3.6	22
50	4.1	3.6	11
60	3.6	3.2	11
70	3.0	3.0	0
80	2.3	2.6	−13
90	1.6	2.0	−25

**Table 3:** Developed neural network (NN) model performance measures for the training, validation, and testing data set.

Data Set	Samples	MSE	R
Training	346,881	0.0171	0.872
Validation	74,331	0.0170	0.872
Testing	74,331	0.0173	0.871

**Table 4:** RMSE values using the AKF and the AKFNN models.

LMP (%)	RMSE (veh)		
	AKF	AKFNN	Improvement (%)
10	4.3	3.7	13
20	4.0	3.6	11
30	3.8	3.5	9
40	3.6	3.3	8
50	3.6	2.7	26
60	3.2	2.4	25
70	3.0	2.4	20
80	2.6	2.3	12
90	2.0	1.8	10

**Table 5:** Impact of applying the trial-and-error technique for the initial value of covariance $P_{i}$.

LMP (%)	RMSE (veh)		
	AKFNN	Tuned AKFNN	Improvement (%)
10	3.7	3.3	11
20	3.6	2.8	22
30	3.5	2.7	23
40	3.3	2.4	27
50	2.7	2.1	22
60	2.4	2.1	13
70	2.4	2.1	13
80	2.3	1.8	22
90	1.8	1.5	17

**Table 6:** RMSE values for the KF, the AKF, the AKFNN, and the tuned AKFNN models.

LMP (%)	RMSE (veh)			
	KF	AKF	AKFNN	Tuned AKFNN
10	6.0	4.3	3.7	3.3
20	5.6	4.0	3.6	2.8
30	5.0	3.8	3.5	2.7
40	4.6	3.6	3.3	2.4
50	4.1	3.6	2.7	2.1
60	3.6	3.2	2.4	2.1
70	3.0	3.0	2.4	2.1
80	2.3	2.6	2.3	1.8
90	1.6	2.0	1.8	1.5
